# Smoking linked to early vision loss and cataracts

**Published:** 2022-11

**20 October 2022** - A new brief finds that smokers stand to develop age-related macular degeneration up to 5.5 years earlier than non-smokers. This blurs a person’s central vision making it difficult for them to do everyday tasks like reading or driving. The brief was developed by WHO, together with the International Agency for the Prevention of Blindness and the University of Newcastle.

People who live with tobacco users are twice as likely to develop age-related macular degeneration from second-hand smoke.

“Smoking increases your risk of developing serious eye conditions and permanent sight loss. Quitting smoking and having regular eye tests can help improve eye health and prevent avoidable sight loss”, said Jude Stern, Head of Knowledge Management, from the International Agency for the Prevention of Blindness.

The brief also highlights that tobacco use increases the risk of developing cataracts. Once cataracts develop, the only way to restore vision is surgical removal and replacement of the cloudy lens. Around 94 million people globally have moderate or severe distance vision impairment or blindness due to unaddressed cataract [1].

E-cigarette flavours may increase the production of free radicals*, which damage DNA, which can lead to cataracts. The use of e-cigarettes may reduce blood flow to the eyes, alter retinal function and lead to an increased risk of developing eye cancer.

“WHO urges everyone not to use tobacco and e-cigarettes to protect their overall health, including eye health,” said Vinayak Prasad, Head of the No Tobacco Unit at the World Health Organization.

Available from: https://www.who.int/news/item/20-10-2022-smoking-linked-to-early-vision-loss-and-cataracts

